# Effectiveness of person-centred versus usual care in elderly patients: findings from a multicentre randomised controlled trial

**DOI:** 10.1080/20523211.2025.2609020

**Published:** 2026-01-19

**Authors:** C. Rovira, M. Casanovas, E. Vizcaino, M. Massanés, Q. Miró, L. Solà, J. Gallego, J. Armengol, C. Ayala, J. Pascual, E. L. Mariño, J. Vidal-Alaball, P. Modamio, PCMR Study Group

**Affiliations:** a Primary and Community Care Management of Central Catalonia, Institut Català de la Salut, Sant Fruitós de Bages, Spain; b Research Group on Health Promotion in Rural Areas, Fundació Institut Universitari per a la recerca a l’Atenció Primària de Salut Jordi Gol i Gurina, Sant Fruitós de Bages, Spain; c Clinical Pharmacy and Pharmaceutical Care Unit, Department of Pharmacy and Pharmaceutical Technology, and Physical Chemistry, Faculty of Pharmacy and Food Sciences, University of Barcelona, Barcelona, Spain; d Chronicity Research Group of Central Catalonia C3RG, University of Vic/Central University of Catalonia, Vic, Spain; e Clinical Pharmacy and Pharmacotherapy Research Group, University of Barcelona, Barcelona, Spain; f Intelligence for Primary Care Research Group, Fundació Institut Universitari per a la recerca a l’Atenció Primària de Salut Jordi Gol i Gurina, Manresa, Spain

**Keywords:** Person-centred care, primary care, multidisciplinary team, frailty, complex chronic patient, polypharmacy, shared decision-making

## Abstract

**Background:**

The person-centred care model involves a multidisciplinary team providing individualised health and social care aligned with patient preferences and health goals. Although some evidence supports its use in primary care, it remains under-evaluated. This study aimed to evaluate the effectiveness of a Collaborative Medication Management (CMM) approach in optimising pharmacotherapy plans versus usual care among polymedicated older adults.

**Methods:**

A randomised, open-label, multicentre, parallel clinical trial was conducted across 11 Primary Care Teams in Spain (Sept 2020–Jan 2024), including patients aged ≥75 years taking ≥8 chronic medications with complex or advanced conditions. The intervention group (IG, *n* = 102) received CMM delivered by a multidisciplinary team, including structured medication review, development of a patient-centred care plan, and follow-up at 6 and 12 months. The control group (CG, *n* = 106) received usual care. Primary outcomes were number of medications, drug-related prescriptions (DRP), changes to pharmacotherapy plans, and hospital admissions. Secondary outcomes included persistence of changes at 12 months and medication-related safety incidents.

**Results:**

A total of 208 subjects were included (65.4% women; mean age 83.6 ± 6.3 years). IG showed a mean reduction of 1.8 ± 2.1 medications/patient vs. an increase of 0.3 ± 1.4 in CG (*p* = 0.004); a mean reduction of 2.9 ± 2.5 DRP/patient in IG vs. An increase of 0.2 ± 1.2 in CG (*p* = 0.004); a mean of 4.3 ± 2.9 changes to pharmacotherapy plans in IG vs. 2.6 ± 3.3 in CG (*p* = 0.004); a mean of 0.3 ± 0.7 hospital admissions in IG vs. 0.3 ± 0.8 in CG (*p* = 1.000). Persistence of changes at 12 months was 3.8 ± 2.7 in IG vs. 2.2 ± 2.9 in CG (*p* < 0.001). In addition, a mean of 0.5 ± 0.7 safety incidents was reported in IG vs. 0.5 ± 1.0 in CG (*p* = 0.676).

**Conclusion:**

The person-centred care model proved effect in optimising medication use in polymedicated older adults.

**Trial registration:** ClinicalTrials.gov identifier: NCT04188470.

## 1. Background

In industrialised countries, the growing ageing population is a key driver of the rise in polypharmacy. Additionally, increased medicalisation, along with advances in diagnosis and treatment, have led to higher rates of comorbidity, polypharmacy, and chronic disease, as well as longer life expectancy. However, these factors have also been associated with more adverse drug events, greater frailty, increased hospital admissions, a decline in quality of life, higher mortality, and rising healthcare costs (Bonano et al., 2025; González-Bueno et al., 2017; Lemstra et al., 2018; Melzer et al., 2015). In Europe, the effect of polypharmacy is widely known. Specifically, in the SIMPATHY (Mair & Fernandez-Llimos, 2017) project, in which most European countries are taking part and whose objective is to establish strategies to reduce polypharmacy, it has already been calculated that there are 8.8 million hospital admissions and/or visits to the emergency department per year due to medication-related adverse events, that 50% of hospital admissions are caused by preventable adverse events, and that 70% of these occur in people over 65 years of age and who are polymedicated. According to a validated scale in the geriatric population (Onder et al., 2010), taking 8 or more medications increases the risk of adverse events fourfold. In the context of pharmacotherapeutic optimisation, it is currently important to talk about excessive polypharmacy, a more qualitative term which, in addition to the number of medications, also includes whether they are necessary and appropriate for the patient.

Excessive polypharmacy is related to a higher risk of inappropriate prescription. In this sense, the literature shows a high percentage of potentially inappropriate prescriptions (PIPs) based on factors such as age, concomitant pathologies, frailty and the complexity of the patients (Campins et al., 2017; Orueta et al., 2015). Despite the existence of multiple prescription support tools, such as lists of medications that are not recommended for use in certain situations due to the risk of adverse events, there is ample room for improvement in the review of the use of these medications in the pharmacotherapy plans of the elderly population. It should be emphasised that the evaluation of therapeutic adequacy in a patient is flexible and dynamic due to the evolution of the pathologies, the frailty and state of the patient, which is why a regular, individualised evaluation of the pharmacotherapy plan with a structured review methodology is essential (Spinewine et al., 2007).

The traditional care model in primary care, in which all responsibility falls on the family doctor and, in some processes, is shared with nursing, entails great care pressure, lack of time and other limitations to assess needs based on greater safety and quality of care. That is why some countries have been incorporating new care roles into primary care in recent years with the aim of offering collaborative work and quality care that responds to the needs of the chronic and complex population.

To mitigate these effects, healthcare organisations have been evaluating and implementing care models that are more complex than the traditional model of single care and sometimes paternalistic care on the part of the doctor. One of these is the person-centred care model, which is based on the coordination of a multidisciplinary team of experts with different knowledge and points of view to offer individualised health and social care and to incorporate the perspective and preferences of the patient, family and/or carer in decisions (Espaulella-Panicot et al., 2017). The Department of Health of Catalonia, one of the regions of Spain with the highest number of primary care pharmacists (PCP), but also with wide variability in the portfolio of services applied, published the person-centred prescribing model in 2022, with the aim of offering appropriate prescribing for frail people with multimorbidity, complex or advanced chronicity. This model includes a systematic and interdisciplinary review of medications in which the clinical pharmacist is included in addition to the medical and nursing professional (Molist-Brunet et al., 2022). Despite some published evidence supporting this model in primary care, to our knowledge, it has not previously been evaluated in our primary care setting. Thus, it would be advisable to have high-quality scientific evidence on the results of this person-centred prescription model in primary care to confirm the model as a strategy for improving therapeutic outcomes. Therefore, this study aimed to evaluate the effectiveness of a multidisciplinary CMM approach that incorporated structured medication review, development of an individualised patient-centred care plan, and follow-up assessments at 6 and 12 months, in improving pharmacotherapy optimisation among polymedicated older adults. To this end, the effectiveness and safety of a person-centred multidisciplinary intervention was compared with usual care in pharmacotherapeutic optimisation. The effectiveness of the intervention was also evaluated in relation to the characteristics of the patients, as well as the persistence of the changes made to the pharmacotherapy plan.

## 2. Methods

A randomised, multicentre, open-label, parallel-group clinical trial was carried out in 11 primary care teams (PCTs) located in central Catalonia, of the Catalan Health Institute (the largest public provider of the Catalan Health Service, the insurer of universal health coverage in Catalonia). The study was registered in the Clinicaltrials.gov database (NCT04188470) and the detailed trial protocol was published prior to the analysis of the results (Rovira et al., 2022). The study was approved by the Ethics Committee for Research with Medication of the Jordi Gol i Gurina Primary Care Research Institute (IDIAPJGol) and also by the Bioethics Commission of the University of Barcelona (CBUB). Informed consent was obtained from all patients or legal representatives before including them in the study. Due to the nature of the intervention, the research team that carried out and evaluated the results of the interventions was not blind to the group assignment. Similarly, it was not possible for the subjects to be blinded. However, the research team that analysed the data was blinded (blind evaluation by third parties).

### 2.1. Recruitment and participants

The subjects included were polymedicated (≥8 drugs with duration >1 month), were ≥75 years old and/or had the status of complex chronic patient (CCP) and/or with advanced chronic disease (PACD) (Santaeugènia et al., 2020). As for CCP, since there is no specific instrument for population screening, the identification was conducted based on expert consensus in the presence of criteria and the results of the three dimensions of complexity, which are summarised in Table 1. Sufficient criteria must have been met for the referring experts to consider that the management of the case was particularly difficult, and, therefore, to validate clinically and identify who was a CCP. Regarding PACD, in Catalonia, the NECPAL CCOMS-ICO © (Gómez-Batiste et al., 2013) instrument was used for the early identification or screening of PACD. This instrument is based on the negative answer (‘I wouldn’t be surprised’) to the question ‘Would you be surprised if this person died over the next year?’, associated with the detection of criteria for palliative needs, functional limitation and/or poor nutritional status, multimorbidity, use of resources and/or criteria of severity and progression of advanced diseases. The definition of one of these two conditions, CCP and PACD, was determined by the basic health unit (BHU) before including the patient in the study through usual care.

**Table 1.** Criteria for the identification of the complex chronic patient (CCP), grouped by domains of complexity.

Clinical status	Multimorbidity or chronic severity pathology Advanced chronic disease Geriatric syndromes Persistent symptoms High utilisation of health services >5% risk by morbidity group
Social status	Social risk
Healthcare system criteria	Benefit from multidisciplinary management Discrepancies among professionals in patient management Benefit in integrated care

Those who had not been monitored by their basic health unit (BHU), consisting of the assigned family doctor and nurse, for at least two visits in the previous year, those who were being monitored by a palliative care programme (PADES), and those who refused to participate in the study were excluded.

### 2.2. Randomisation and sampling

Stratified randomisation was performed at the level of PCTs, based on their geographical location. A total of 12 PCTs were randomly assigned (1:1) to either the control group (CG) or the intervention group (IG) using a computer-generated randomisation sequence. Within each PCT, BHUs that voluntarily agreed to participate were automatically assigned to the same study group as their corresponding PCT. Subsequently, patients aged ≥75 years assigned to participating BHUs were randomly selected (1:1) from a list of eligible individuals extracted from the Catalan Health Institute database. This selection was also performed using computer-generated random numbers. Each patient was thus assigned to the CG or IG according to the BHU to which they belonged. One PCT did not recruit any patients and was excluded from the final analysis. Consequently, the results from 11 PCTs were analysed.

### 2.3. Trial procedure

#### 2.3.1. Intervention group

The intervention consisted of implementing the person-centred care model (Molist-Brunet et al., 2022) through a multidisciplinary team composed of a family physician, a nurse and a clinical pharmacist. This team applied a Collaborative Medication Management (CMM) approach, which included a structured medication review, development of patient-centred care plan, and follow-up visits at 6 and 12 months to optimise the pharmacotherapy plan. Specifically, the first stage consisted of a multidimensional evaluation of the subject using comprehensive geriatric assessment and frailty scales and a review of the pathologies and the degree of their control; the therapeutic objectives of the subject were then agreed in such a way that in the most frail, symptom control and well-being and comfort were prioritised, in those of intermediate frailty, the stable maintenance of functionality was prioritised and, finally, in the least frail, the objective of disease prevention was chosen. The second stage involved applying a medication review methodology developed by the Spanish Society of Primary Care Pharmacists (SEFAP) (Amado et al., (2012))). In this stage, patient pathologies were prioritised according to severity and risk of complications, and linked to the prescribed medications. Subsequently, a therapeutic adequacy algorithm was applied to each medication, reviewing indication, dose, regimen, duration, safety and whether the patient collected it from the community pharmacy. These recommendations were used to update the pharmacotherapy plan, which in the third stage was agreed upon with the patient and/or carer through a shared decision-making process. Finally, follow-up was carried out with visits at 6 and 12 months.

#### 2.3.2. Control group

The usual clinical practice was carried out, which includes the healthcare by the family physician and/or nurse, together with the usual support of the PCP and the use of prescription support tools incorporated into the primary care computerised medical record. This support consisted of activities aimed at improving prescribing quality, such as monitoring prescribing indicators, providing educational sessions, issuing standard recommendations, and generating patient lists with potentially inappropriate prescriptions or non-recommended medications. No structured medication review or patient-centred care plan was performed in this group.

### 2.4. Data collection

Data was collected at 0, 6 and 12 months through the computerised medical records of the subjects included. For this purpose, a data collection notebook was designed to be filled in by the PCP and a database (Excel). All study data were coded, and their confidentiality was guaranteed.

### 2.5. Outcomes

To accurately measure therapeutic optimisation and given the complexity of doing so with a single outcome, the effectiveness of the intervention was evaluated using four co-primary outcomes that included the variation in the mean number of medications prescribed, variation in the mean number of drug-related problems (DRPs), variation in the mean number of changes implemented in pharmacotherapy plans, and hospital admissions. A drug-related problem (DRP) was defined as any event or circumstance involving drug therapy that actually or potentially interferes with desired health outcomes (PCNE classification., 2020). Specifically, this category included PIPs, defined as any medication considered unsuitable for the patient according to explicit criteria (e.g. STOPP/START, Beers), taking into account age, comorbidities, and treatment goals (O’Mahony et al., 2023; American Geriatrics Society, 2023 Updated AGS Beers Criteria® for Potentially Inappropriate Medication Use in Older Adults, 2023)

The secondary outcomes were the persistence of the mean number of changes in the pharmacotherapy plan at 12 months and the variation in the mean number of medication-related safety incidents, comprising adverse drug reactions (ADR) and DRPs resulting from modifications in the pharmacotherapy plan, detected at 6 and 12 months.

All these outcomes were also analysed by gender. Additionally, we assessed the frequency and mean variation of each DRP type, the frequency of each type of change implemented in pharmacotherapy plans, and each type of medication-related safety incident detected. The severity of medication-related safety incidents was classified according to the level of healthcare required. Incidents that resulted in an emergency department visit, hospital admission, or death were categorised as severe; all remaining events were considered non-severe. Worsening of symptoms following deprescribing or dose adjustment was included in this severity framework. For each non-severe incident, we recorded the corresponding management actions including medication reintroduction, dose modification, initiation of additional therapies, or drug discontinuation over the 12-month follow-up period.

### 2.6. Statistical analysis

The sample size was calculated using the GRANMO software (version 7.12), applying a two-proportion comparison for independent groups. Assuming a two-sided alpha risk of 0.05, a beta risk of 0.20, and an anticipated dropout rate of 15%, a minimum of 103 subjects per group was required to detect a statistically significant difference in the mean reduction of hospital admissions per patient. Based on preliminary estimates, the expected reduction was 0.18 admissions per patient in the IG and 0.05 in the CG.

Given that the study included four main covariates in the analysis, the Bonferroni correction method was applied to adjust for multiple comparisons, thereby ensuring the robustness of the statistical significance threshold.

A descriptive analysis was performed for all outcome variables. For qualitative outcomes, frequencies and percentages were calculated. For quantitative outcomes, means and standard deviations (SDs) were computed. Bivariate analyses were conducted to compare outcomes between groups. For variables with two categories, Student’s t-test was used; for variables with more than two categories, one-way ANOVA was applied.

To assess the influence of potential confounding factors on the four co-primary outcomes, a multiple regression analysis was performed. Continuous outcomes were analysed using multiple linear regression, which estimates β coefficients, while binary or count outcomes were analysed using generalised linear models, including binomial and Poisson families. The following were considered potential confounding variables: gender, environment (rural/urban), carer, presence of a chronic and complex condition, inclusion in the home care programme (ATDOM), residence in a nursing home, high risk of hospital readmission, and frailty. The statistical software R version 4.2.1 (R Foundation for Statistical Computing) was used for all analyses. All analyses were conducted per-protocol population, excluding participants who did not complete at least 6 months of follow-up, to ensure that the analysis reflected the effect of the intervention among participants who adhered to the study procedures, and data from patients who dropped out of the study were excluded from the final analysis.

### 2.7. Patient participation

No patients were involved.

## 3. Results

A total of 210 subjects were included in the trial between July 2020 and January 2023. Of these, practically all, 208 (83.6 ± 6.3 years, 65.4% women), completed the 12-month follow-up. 102 subjects were assigned to the IG and 106 to the CG. Follow-up of the last subject included was completed in January 2024. Table 2 shows the baseline characteristics of the subjects included.

**Table 2.** Descriptive study of the sample.

	Control	Intervention	*p*-value
	*n* = 106	*n* = 102	
Age, mean (SD)	83.3 (5.9)	84.3 (6.5)	0.241
Gender (M = 0, F = 1), *n* (%)			0.120
0	42 (39.6%)	29 (28.4%)	
1	64 (60.4%)	73 (71.6%)	
Type of PCT (urban = 0, rural = 1), *n* (%)			0.192
0	30 (28.3%)	20 (19.6%)	
1	76 (71.7%)	82 (80.4%)	
Carer (No = 0, Yes = 1), *n* (%)			0.821
0	41 (38.7%)	42 (41.2%)	
1	65 (61.3%)	60 (58.8%)	
Chronicity 0 (No = 0, CCP = 1, PACD = 2), *n* (%)			**<0.001**
0	57 (53.8%)	29 (28.4%)	
1	41 (38.7%)	69 (67.6%)	
2	8 (7.6%)	4 (3.9%)	
ATDOM programme (No = 0, Yes = 1), *n* (%)			**0.017**
0	92 (86.8%)	74 (72.5%)	
1	14 (13.2%)	28 (27.5%)	
Nursing home (No = 0, Yes = 1), *n* (%)			**<0.001**
0	95 (89.6%)	63 (61.8%)	
1	11 (10.4%)	39 (38.2%)	
Risk of readmission, mean (SD)	12.9 (7.52)	11.6 (6.37)	0.173
Frailty index, mean (SD)	0.28 (0.16)	0.36 (0.15)	**0.001**

SD: Standard deviation; M: Male; F: Female; PCT: Primary care team; CCP: Complex chronic patient; PACD: Patient with advanced chronic disease; ATDOM: Home care. Bold values indicate statistically significant differences (*p* < 0.05).

### 3.1. Primary outcomes

**Figure 1.**
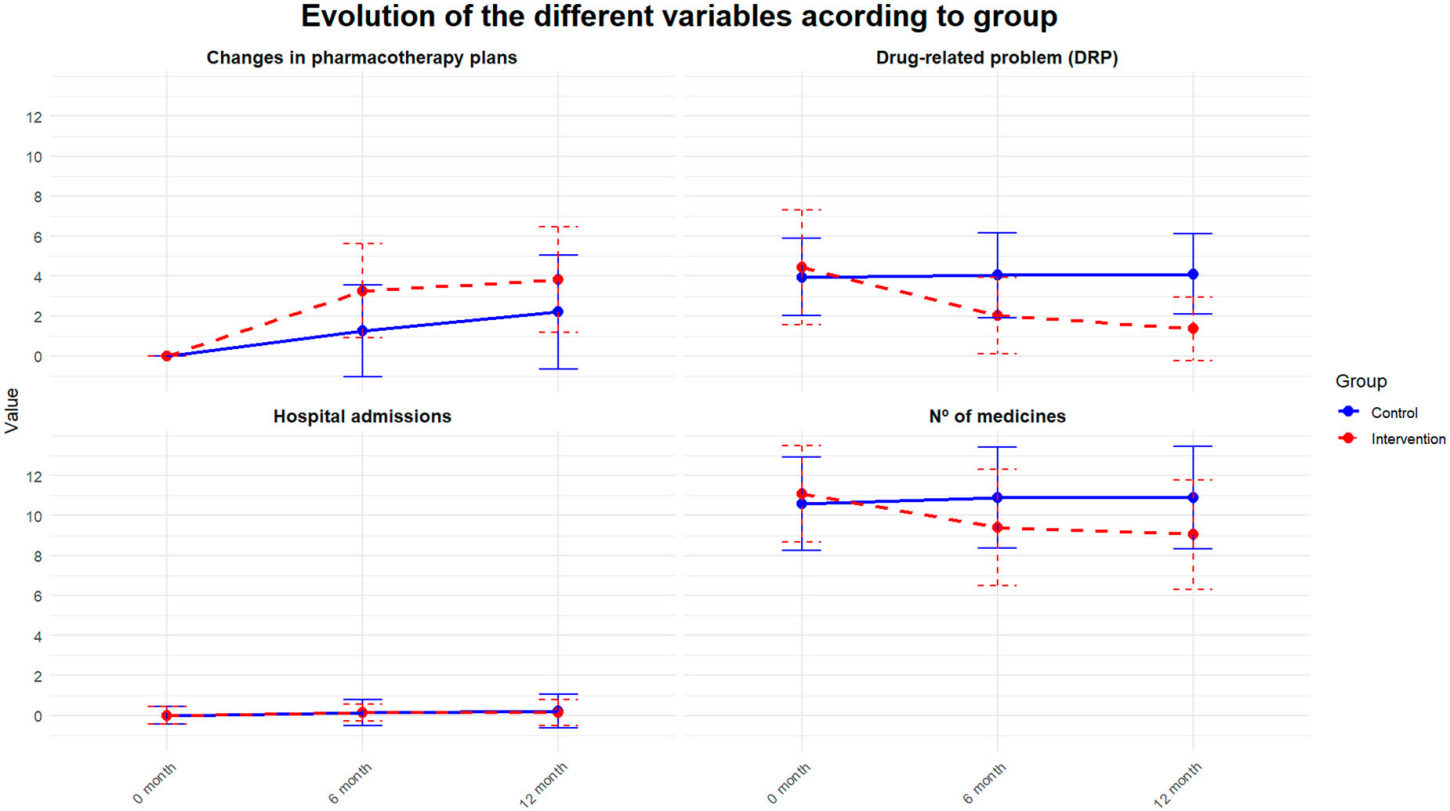
Results of the main variables in the control group and in the intervention group. This figure shows the results of the main variables of the study at 0, 6 and 12 months for each group in the study. The Y-axis ‘value’ measures the mean of each variable and the X-axis the time variable in which each variable has been measured. In the intervention group, the difference in all the variables at each point in time can be clearly seen, except for hospital admissions. In contrast, in the control group, the small difference in all the variables at each point in time can be clearly seen, except for the number of changes made to the pharmacotherapy plan.

Figure 1 shows the differences in the variation of the mean of each co-primary outcome at 0, 6 and 12 months for each study group.

Significant differences in favour of the IG were observed in the variation of the mean of the number of medications prescribed, the number of DRPs and the number of changes made to the pharmacotherapy plan at both 6 and 12 months. In contrast, no significant differences were observed in the mean number of hospital admissions (Table 3).

**Table 3.** Description of results of the study outcomes at 12 months.

Outcomes	Results			
Co-primary outcomes	Control	Intervention	*p*-value	Adjusted *p*-value*
Δ DRP, mean (SD)	0.16 (1.20)	−2.93 (2.47)	**<0.001**	**0.004**
Δ Number of medications prescribed, mean (SD)	0.32 (1.44)	−1.79 (2.14)	**<0.001**	**0.004**
Changes made to pharmacotherapy plan, mean (SD)	2.55 (3.28)	4.31 (2.87)	**<0.001**	**0.004**
Hospital admissions, mean (SD)	0.33 (0.84)	0.27 (0.65)	0.541	1.000
Secondary outcomes	**Control**	**Intervention**	***p*-value**	
Changes made to pharmacotherapy plan that are maintained at 12 months, mean (SD)	2.21 (2.86)	3.83 (2.65)	**<0.001-**	
Safety incidents, mean (SD)	0.52 (1.00)	0.47 (0.70)	0.676-	

SD: Standard deviation; DRP: Drug-related problem; (*): Adjusted *p*-value by Bonferroni method. Bold values indicate statistically significant differences (*p* < 0.05).

Table 4 shows the results of the study outcomes segregated by gender. Similar results were found in the analysis without considering the gender of the participants, with no differences between genders for each outcome.

**Table 4.** Description of result variables according to gender.

Variables	Women			Men		
	Control	Intervention	*p*-value	Control	Intervention	*p*-value
Mean number of medications prescribed at 0 months (SD)	10.3 (2.13)	11.0 (2.50)	0.108	11.0 (2.60)	11.3 (2.20)	0.701
Mean number of medications prescribed at 12 months (SD)	10.5 (2.51)	9.00 (3.01)	**0.002**	11.5 (2.55)	9.16 (1.91)	**<0.001**
**Δ Mean number of medications prescribed (SD)**	0.20 (1.53)	−1.81 (2.06)	**<0.001**	0.50 (1.30)	−1.76 (2.39)	**<0.001**
Mean DRP at 0 months (SD)	3.98 (1.85)	4.39 (2.45)	0.276	3.90 (2.07)	4.57 (3.76)	0.386
Mean DRP at 12 months (SD)	3.98 (1.89)	1.22 (1.46)	**<0.001**	4.28 (2.18)	1.72 (1.84)	**<0.001**
**Δ Mean DRP 0–12 months (SD)**	0.03 (1.02)	−3.07 (2.32)	**<0.001**	0.35 (1.42)	−2.56 (2.84)	**<0.001**
**Mean number of changes to the pharmacotherapy plan (SD)**	2.90 (3.81)	4.25 (2.63)	**0.020**	2.00 (2.11)	4.47 (3.44)	**0.001**
**Mean hospital admissions (SD)**	0.31 (0.80)	0.25 (0.58)	0.650	0.38 (0.90)	0.32 (0.75)	0.791
**Mean number of changes made to the pharmacotherapy plan that are maintained at 12 months (SD)**	2.49 (3.30)	3.78 (2.28)	**0.011**	1.77 (1.95)	3.96 (3.54)	**0.008**
**Mean number of safety incidents (SD)**	0.48 (1.02)	0.57 (0.79)	0.587	0.58 (0.98)	0.23 (0.43)	0.057

SD: Standard deviation; DRP: Drug-related problem. Bold values indicate statistically significant differences (*p* < 0.05).

The most frequently type of DRP detected were inappropriate dose or regimen, inappropriate duration, unsuitable medication, therapeutic intensity inappropriate for frailty, inefficient alternative, discouraged medication and therapeutic duplication. Differences were observed in the detection of the type of DRP between groups, such that in the IG more DRPs related to unsuitable dose or regimen and inadequate therapeutic intensity according to frailty were detected; in contrast, more DRPs related to inefficient alternatives were detected in the CG (Table 5).

**Table 5.** Analysis of types of drug-related problem (DRP).

Types of DRP	0 months			12 months			Variation	
	Control (%)	Intervention (%)	*p*-value	Control (%)	Intervention (%)	*p*-value	Control (%)	Intervention (%)
Inadequate dosage/regimen	40.0	59.8	**0.007**	38.0	20.7	**0.014**	−5	−65.3
Therapeutic duplicity	15.2	18.6	0.642	14.0	8.70	0.354	−7.89	−53.2
Alert and/or contraindication	15.2	12.7	0.752	15.0	4.35	**0.026**	−1.31	−65.7
Avoidable medication	9.52	11.8	0.766	10.0	2.17	0.052	5.04	−81.6
Inappropriate duration	60.0	48.0	0.113	58.0	28.6	**<0.001**	−3.33	−40.0
Risk combinations	5.71	2.94	0.498	11.0	0.00	**0.003**	92.6	−100
Discouraged medication	25.7	30.4	0.552	25.0	19.6	0.466	−2.72	−35.5
Inefficient alternative	55.2	40.2	**0.043**	52.0	13.0	**<0.001**	−5.79	−67.6
Unapproved medication	52.4	46.1	0.443	57.0	14.1	**<0.001**	8.77	−69.4
Inadequate therapeutic intensity	12.4	41.2	**<0.001**	13.0	5.43	0.121	4.83	−86.8
Therapeutic cascade	1.90	0.98	1.000	2.00	0.00	0.498	5.26	−100
Necessary medication	3.81	9.80	0.150	8.00	1.09	**0.036**	109	−88.8

Bold values indicate statistically significant differences (*p* < 0.05).

At 12 months, all types of DRP decreased in the IG, the incidents that decreased the most (>80%) were therapeutic cascades, risk combinations, necessary medication not prescribed, inadequate therapeutic intensity, and avoidable medication. In the CG, 6 types of incidents decreased (therapeutic duplicity, inefficient alternative, inappropriate dose or regimen, inappropriate duration, discouraged medication, and alert and/or contraindication). It should be noted that the decrease was in most cases <10%, and that all other incidents increased (Table 5).

In the IG, more changes were made to the pharmacotherapy plan than in the CG. On the other hand, in the IG most changes took place during the first 6 months (3.3 (SD 2.4) versus 1.3 (SD 2.3) in the CG; *p* = 0.004) (Figure 1). Significant differences were observed between the two groups in the percentage of patients who underwent some change to the pharmacotherapy plan, such that in the IG at least one drug was withdrawn in 78.4% of subjects while in the CG it was withdrawn in 47% (*p* < 0.001). The dose of some medication was changed in 57.7% of the subjects in the IG and in 17% in the CG (*p* < 0.001) and a new drug was introduced in 27.8% of the subjects in the IG and in the CG it was 49% (*p* = 0.004). Table 6 shows all the data related to the type of change made in the pharmacotherapy plan.

**Table 6.** Frequency (%) of subjects for each type of change made.

Type of change to pharmacotherapy plan by study group	Control (%)	Intervention (%)	*p*-value
Withdrawal of drug	47	78.4	**<0.001**
Change of dose	17	57.7	**<0.001**
Change of regimen	5	10.3	0.256
Change of drug	18	30.9	0.052
New drug	49	27.8	**0.004**

Bold values indicate statistically significant differences (*p* < 0.05).

In the analysis of the four co-primary outcomes, a multiple regression analysis was conducted to adjust for potential confounding outcomes such as gender, environment (rural/urban), carer, chronicity status, inclusion in the ATDOM programme, nursing home residence, risk of readmission, and frailty. Table 7 shows the adjusted effects of the intervention and other covariates on the four co-primary outcomes: variation in the number of medications, DRP variation, persistence of changes made to the pharmacotherapy plan, and number of hospital admissions. It was observed that having a carer and being at greater risk of readmission were significantly associated with a greater number of changes to the pharmacotherapy plan that were maintained at 12 months (*p* = 0.012). However, no other significant associations were observed (Table 7).

**Table 7.** Multivariate analysis.

	Variation in the number of medications			DRP variation			Variation of changes made that are maintained after 12 months			Number of admissions		
	Coefficient	*p*-value	Adjusted *p*-value*	Coefficient	*p*-value	Adjusted *p*-value*	Coefficient (RR)	*p*-value	Adjusted *p*-value*	Coefficient (RR)	*p*-value	Adjusted *p*-value*
**Intercept**	0.034	0.992	1.000	−2.316	0.280	1.000	1.409	0.577	1.000	1.546	0.823	1.000
**Study group**												
Control	Ref.			Ref.			Ref.			Ref.		
Intervention	−2,709	**<0.001**	**0.012**	−3.425	**<0.001**	**0.012**	1,787	**<0.001**	**0.012**	0.759	0.362	1.000
Age, *median (SD)	0.019	0.644	1.000	0.035	0.179	1.000	0.996	0.614	1.000	0.970	0.211	1.000
**Gender (M** **=** **0, F** **=** 1), *n (%)												
0	Ref.			Ref.			Ref.			Ref.		
1	0.574	0.275	1.000	−0.168	0.602	1.000	1,288	**0.007**	0.084	0.976	0.932	1.000
**Type of PCT (rural = 1, urban = 0)**												
0	Ref.			Ref.			Ref.			Ref.		
1	−0.172	0.782	1.000	0.363	0.343	1.000	1.263	**0.048**	0.576	0.639	0.194	1.000
**Carer (No** **=** **0, Yes = 1)**												
0	Ref.			Ref.			Ref.			Ref.		
1	−0.226	0.692	1.000	−0.945	**0.007**	0.084	1,541	**<0.001**	**0.012**	1,344	0.369	1.000
**Chronicity (No** **=** **0, CCP = 1, PACD = 2)**												
0	Ref.			Ref.			Ref.			Ref.		
1	−0.358	0.592	1.000	−0.097	0.812	1.000	0.859	0.187	1.000	2,002	0.064	0.768
2	−2,247	0.050	0.600	−0.056	0.936	1.000	0.954	0.809	1.000	1,050	0.937	1.000
**ATDOM programme (No** **=** **0, Yes = 1)**												
0	Ref.			Ref.			Ref.			Ref.		
1	1,320	0.084	1.000	0.690	0.141	1.000	1,316	**0.027**	0.324	1,120	0.774	1.000
**Nursing home**												
0	Ref.			Ref.			Ref.			Ref.		
1	0.368	0.627	1.000	0.380	0.414	1.000	0.913	0.483	1.000	0.996	0.991	1.000
**Risk of readmission**	−0.036	0.346	1.000	−0.010	0.656	1.000	1,027	**<0.001**	**0.012**	1,050	**0.008**	0.096
**Level of frailty**	−5,062	**0.010**	0.120	−0.621	0.607	1.000	0.486	**0.034**	0.408	0.988	0.991	1.000

SD: Standard deviation; M: Male; F: Female; DRP: Drug-related problem; PCT: Primary care team; CCP: Complex chronic patient; PACD: Patient with advanced chronic disease; ATDOM: Home care; (*): Adjusted *p*-value by Bonferroni method. Bold values indicate statistically significant differences (*p* < 0.05).

Regarding the outcome of number of hospital admissions, no significant differences were found between the two groups. However, throughout the study a greater favourable difference was observed for the IG as changes were made to the pharmacotherapy plan, being from 0 to 6 months for IG 0.1 (SD 0.4) versus CG 0.1 (SD 0.4), *p* = 1.000; and from 6 to 12 months for IG 0.1 (SD 0.4) versus CG 0.2 (SD 0.64), *p* = 0.856; it should be noted that in the CG some patients suffered up to 5 admissions in 12 months. No relationship was observed with the confounding variables (Table 7).

### 3.2. Secondary outcomes

Table 3 also shows the distribution of the results of the secondary outcomes. Significant differences were observed in the persistence of the changes at 12 months: in the IG a mean of 3.8 changes in the pharmacotherapy plan were maintained and in the CG a mean of 2.2 changes (*p* < 0.001). It should be noted that >80% of the changes made in both study groups were maintained at 12 months.

In terms of medication safety, a mean of 0.5 (SD 0.7) safety incidents were reported in the IG and 0.5 (SD 1.0) in the CG (*p* = 0.676), with no significant differences observed between the two groups. Severe safety incidents occurred in 7.8% of patients in the CG compared with 3.0% in the IG (*p* = 0.214; risk ratio = 0.39). Although not statistically significant, this difference indicates a clinically important reduction in severe events among those receiving the intervention.

When examining incidents by type (Table 8), 25.8% of patients in the IG experienced symptom worsening, yet none of these episodes were classified as severe. These findings are likely associated with the withdrawal of benzodiazepines and proton pump inhibitors, the most common deprescribing actions in the IG (Table 6), compared with only 4% undergoing similar changes in the CG (*p* < 0.001).

**Table 8.** Frequency (%) of each type of safety incident detected.

Type of safety incident	Control (%)	Intervention (%)	*p*-value
Withdrawal syndrome	0	0	.
Rebound effect	0	3.09	0.117
Worsening of symptoms	4	25.8	**<0.001**
Medication error	2	0	0.498
Emergency care	8	2.06	**0.035**
Hospital admission	0	1.03	0.241
Exitus	0	0	0.492
Medication-related adverse events	25	11.3	**0.022**

Bold values indicate statistically significant differences (*p* < 0.05).

On the other hand, in the IG, 2.1% of subjects required urgent attention for medication-related adverse events compared to 8% of subjects in the CG (*p* = 0.035). Additionally, 11.3% of subjects in the IG suffered a medication-related adverse event that did not require urgent attention compared to 25% of subjects in the CG (*p* = 0.022). Furthermore, the same drug was reintroduced in 13.4% of cases in the IG versus 2% in the CG (*p* = 0.006) (Tables 8 and 9).

**Table 9.** Frequency (%) of actions as a result of detected safety incidents.

Type of post-safety incident action	Control (%)	Intervention (%)	*p*-value
Reintroduction of drug	2	13.4	**0.006**
New drug	3	9.28	0.123
Change of dose	9	7.22	0.844
Withdrawal of drug	5	3.09	0.721

Bold values indicate statistically significant differences (*p* < 0.05).

## 4. Discussion

This clinical trial comparing the effectiveness of the collaborative medication management as a medication review model versus usual care. The one carried out by a multidisciplinary team made up of family medicine and community health professionals, primary care nurses and clinical pharmacists and sometimes other specialists, using a structured process and in accordance with the person-centred review methodology, offers, as similar studies have shown (Campins et al., 2017; Kua et al., 2019; Syafhan et al., 2021; Tan et al., 2014), positive results in terms of therapeutic optimisation and health benefits for patients with comorbidity and complexity (McKee et al., 2019). A person-centred review of the prescription significantly reduces the number of medications and the number of DRPs and even manages to maintain the changes made persistently for at least 12 months.

On the other hand, the incorporation of the clinical pharmacist in the pharmacotherapy plan review process in polymedicated patients in primary care manages to reduce prescription errors and adverse events (Hughes et al., 2019; Pellegrino et al., 2009) given that, as an expert in the use of medications, they contribute to their identification, in addition to providing an individualised and optimised pharmacotherapy plan by reviewing the need, effectiveness and appropriateness of each medication, thus adjusting doses or regimens and selecting safer and more effective alternatives, as was also observed in the IG of this clinical trial.

However, unlike other clinical trials (Hung et al., 2024) carried out in different settings such as hospitals, social-healthcare centres and even in nursing homes, this clinical trial carried out in the primary care setting did not demonstrate statistical significance in the reduction of hospital admissions. Although the sample size was calculated to detect a difference in hospital admissions, the observed values did not reflect the expected effect size. Therefore, while the sample size assumptions were met, the lack of statistical significance in this outcome may be due to the actual effect being smaller than anticipated, rather than insufficient power. The patients included in this trial are complex, they suffer from chronic conditions that require regular care, sometimes from professionals at different levels of care, they are vulnerable to acute exacerbations that are difficult to predict and that can lead to hospitalisation regardless of the optimisation applied in the pharmacotherapy plan. Moreover, lack of adherence due to social, cultural or other factors limits the effectiveness of preventing hospital admissions. Along these lines, the systematic review by Croke et al. (2023) concluded that the incorporation of a clinical pharmacist in primary care reduces the number of DRPs and the number of medications prescribed, but the results in terms of improvement in quality of life were not conclusive due to the heterogeneity and lack of standardisation of this type of intervention.

Although this study aims to evaluate the impact on pharmacotherapeutic adequacy in polymedicated patients, it also measured the decrease in the number of medications prescribed. There are multiple studies (Hung et al., 2024) that evaluate the effects of deprescribing in elderly and polymedicated populations, but the interventions are heterogeneous, such that some include only a review by the clinical pharmacist and a proposal of recommendations, achieving a significant reduction in DRPs in the IG, but not in the use of healthcare resources (Lenander et al., 2014). Other interventions measure the use of prescription support tools commonly incorporated into the clinical workstation (Fried et al., 2017; Jungo et al., 2023). If we compare the results of this clinical trial with others that measure the effectiveness of using prescription support tools and systems to warn of inappropriate prescriptions through the clinical station versus not using them, these did not demonstrate effectiveness in reducing DRPs or hospital admissions. Therefore, the model studied offers advantages over other interventions.

With regard to the persistence of changes in the pharmacotherapy plan, it was high in both groups (> 80%), however, favourable differences were observed for the IG. Although there are few studies (Campins et al., 2017) measuring the persistence of changes, in the study by Mortsiefer et al. (2023) in which the medication review intervention focused on the shared decision-making process, significant differences were observed in the reduction of DRPs at 6 months, but not at 12 months between the two study groups. However, in the model of this clinical trial, the differences in the changes at 12 months were maintained. Although the reduction in severe incidents did not reach statistical significance (*p* = 0.214), the intervention group exhibited a clinically meaningful decrease in events requiring urgent care or hospitalisation. These findings suggest that person-centred medication review may improve patient safety, even if the study lacked sufficient power to detect differences in rare severe outcome.

Regarding medication-related adverse events, both in those requiring urgent attention and those that did not were significantly more frequent in the control group, particularly common events such as falls and hypoglycaemia. These findings indicate that the reduction in medications and DRPs observed in the IG could have a protective effect against medication-related adverse events. In contrast, the IG suffered a greater number of cases of worsening symptoms due to pharmacotherapy plan changes; however, these did not require hospital or emergency care and were mostly resolved by reintroducing the withdrawn medication.. On the other hand, the intervention proved to be safe as it did not increase the incidence of safety events compared to usual clinical practice. The impact in terms of patient safety has been widely documented in other published studies that show a reduction in the number of falls, sedative effects and cognitive impairment (Mahlknecht et al., 2021; Van der Meer et al., 2018).

In view of the above, the intervention studied is in line with studies showing that the systematic review of medication by a multidisciplinary team can help to identify DRPs, optimise doses and regimens, as well as propose safer alternatives, which contributes to reducing the risk of medication-related adverse events (Poudel et al., 2016; Vinks et al., 2018).

## 5. Limitations and strengths

Among the strengths of this clinical trial, which investigates the effect of a multidimensional intervention (frailty, clinical complexity, morbidity and therapeutic adequacy), is the fact that it has been carried out in a real primary care clinical setting and with an innovative multidisciplinary collaborative work in Spain as it incorporates the figure of the primary care clinical pharmacist. PCPs participate in this comprehensive care, contributing their specialised knowledge of medications. Another strength is that the intervention is based on person-centred care, which aims to improve the decision-making capacity and autonomy of polymedicated patients aged 75 and over. The 1-year follow-up made it possible to measure the effects of the intervention over time.

Despite this, the difficulty in organisational management when developing this model was a limitation in the healthcare environment as this multidimensional methodology requires time and adequate coordination between team members. On the other hand, the fact that it is an inevitably open study in usual clinical practice and that patients and some professionals may be reluctant to modify chronic treatments prescribed for years, may have led to the appearance of possible biases. The non-randomisation of the participating BHUs, as only those that wished to participate were included, represented a selection bias, as some of the participating BHUs specifically treated patients in ATDOM programmes or who resided in nursing homes, and as is well known, these patients are more complex and frailer. However, it is precisely these patients who are the target population for the application of the person-centred review model. The analysed follow-up period of 12 months was insufficient to reduce hospitalisations in polymedicated patients, probably due to their clinical complexity and frailty.

As for the per-protocol analysis, this decision was made because a proportion of participants, due to advanced age and clinical complexity, died before completing the 6-month follow-up, and including these cases in the main analysis could have introduced bias in assessing the intervention’s effects; however, using this per-protocol approach may also introduce selection bias and limit the generalisability of the findings, which should be taken into account when interpreting the results.

Finally, this study is limited by the lack of patient involvement in its design, conduct, or interpretation, apart from participation in the clinical trial. Future studies should actively integrate patient and public involvement strategies to enhance the relevance, applicability, and overall impact of the findings in primary care.

## 6. Conclusion

This clinical trial evaluated the impact of collaborative work within a multidisciplinary team that incorporated a primary care pharmacist with a clinical role to implement a patient-centred care model in routine practice, with the aim of optimising therapeutic plans for elderly polymedicated patients. Although similar collaborative medication-management approaches have been adopted in primary care settings elsewhere, this study represents one of the first implementations of such a model in Spain, integrating a clinically active primary care pharmacist into the team. The intervention demonstrated reductions in polypharmacy, improvements in pharmacotherapeutic adequacy and a better safety profile for medication use in this population. These findings reinforce evidence that multidisciplinary approaches to pharmacotherapy optimisation provide significant benefits for older adults. Finally, in order to advance research, a cost-utility study is planned to assess the feasibility of implementing the model.

## 7. Relevance and applicability

There is published evidence (Spinewine et al., 2007; Tan et al., 2014) on the result of the implementation of these interventions, but in the field of primary care they are scarce, so this clinical trial aims to expand knowledge on the subject.

**List of abbreviations**
ADR: adverse drug reactions
ATDOM: Home care
BHU: Basic health unit
CBUB: Bioethics Commission of the University of Barcelona
CCP: Complex chronic patient
CG: Control group
CMM: Collaborative Medication Management
DRP: Drug-Related Problems
ECAP: Primary care clinical workstation
ESCA: Health Survey of Catalonia
IDIAP JGol: Jordi Gol i Gurina Primary Care Research Institute
IG: Intervention group
PACD: Patient with advanced chronic disease
PADES: Palliative care programme
PCMR: person-centred medication review
PCP: Primary care pharmacist
PCT: Primary Care Team
PIP: Potentially inappropriate prescription
SEFAP: Spanish Society of Primary Care Pharmacists
SD: Standard deviation

## Availability of the data and materials

The study generated several Excel documents that will be kept only for the duration of the study and will be in the custody of the principal researcher. These documents are also password protected. The database is not available but can be obtained by request through the corresponding author.
